# Acetaminophen-Induced Type II Kounis Syndrome: A Case Report Highlighting Diagnostic Challenges and the Utility of a Physician-Staffed Rapid Response Car

**DOI:** 10.7759/cureus.104346

**Published:** 2026-02-26

**Authors:** Satoshi Hashiguchi

**Affiliations:** 1 Emergency Medicine, Tokushima Red Cross Hospital, Tokushima, JPN

**Keywords:** acetaminophen, anaphylaxis, intensive care unit stay, kounis syndrome, medical intensive care unit (micu), rapid response car

## Abstract

Kounis syndrome (KS) is a clinical condition where anaphylaxis induces acute coronary syndrome. This report describes a case of Type II KS in an 85-year-old male, triggered by intravenous acetaminophen, resulting in total occlusion of the right coronary artery. The intervention of a physician-staffed rapid response car (RRC) facilitated early pre-hospital diagnosis of ST-segment elevation myocardial infarction (STEMI). This enabled an integrated transition to emergency percutaneous coronary intervention (PCI). The case demonstrates the utility of advanced pre-hospital systems in managing the therapeutic conflicts associated with KS.

## Introduction

Kounis syndrome (KS), first identified in 1991, represents a complex cardiovascular condition defined as the coincidence of acute coronary syndromes, ranging from vasospastic angina to acute myocardial infarction, associated with mast cell and platelet activation during hypersensitivity reactions [1]. The pathophysiology involves the release of inflammatory mediators such as histamine, tryptase, and leukotrienes, which can induce coronary artery vasospasm or lead to plaque erosion and rupture [2]. While a wide array of pharmacological agents, environmental exposures, and medical conditions have been identified as triggers, intravenous acetaminophen remains an infrequently reported cause. Acetaminophen is widely regarded as a safe analgesic; however, rare instances of hypersensitivity leading to coronary events have been documented, emphasizing the need for clinical vigilance [3]. Despite its clinical significance, KS is often underdiagnosed because the dramatic systemic manifestations of anaphylaxis may mask the signs of cardiac ischemia. Furthermore, the management of KS presents a unique "therapeutic paradox," as the administration of epinephrine for anaphylaxis can exacerbate myocardial oxygen demand and worsen coronary vasospasm [4]. This report evaluates a case of acetaminophen-induced Type II KS and highlights the pivotal role of a physician-staffed rapid response car (RRC) in facilitating early diagnosis and navigating the complex therapeutic pathway.

## Case presentation

Initial presentation and pre-hospital care

An 85-year-old male with a history of hypertension presented to a primary care physician with fever and vertigo. Intravenous acetaminophen was administered for febrile relief. Immediately following administration, the patient developed hypotension and dyspnea. Suspecting anaphylactic shock, the primary care physician administered 0.3 mg of intramuscular epinephrine twice, resulting in transient stabilization of blood pressure.

Due to the severity of the reaction, a physician-staffed RRC was dispatched. Upon arrival of the RRC physician, the patient's vitals were blood pressure 126/74 mmHg, heart rate 60 bpm, and oxygen saturation 100% on 4 L/min nasal oxygen. Respiratory distress and wheezing had subsided, but the patient reported intense retrosternal chest pain during transport.

Cardiac event and acute management

**Figure 1:**
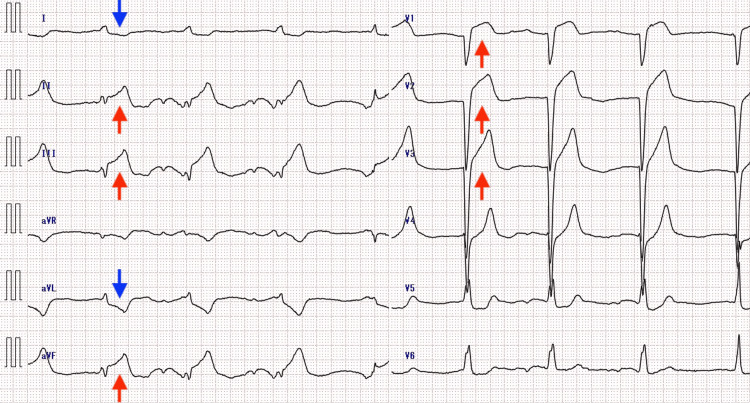
12-lead electrocardiogram (ECG) recorded by the rapid response car team. The ECG shows a significant ST-segment elevation in the inferior leads (II, III, and aVF) and reciprocal ST-depression in leads I and aVL. Solid arrows indicate ST-segment elevation, which is also observed in leads V1–V3, suggesting concurrent right ventricular infarction.

A pre-hospital 12-lead electrocardiogram (ECG) performed by the RRC team revealed ST-segment elevation in the inferior leads (II, III, aVF) with reciprocal changes in leads I and aVL. The RRC physician diagnosed KS and notified the receiving hospital to activate the catheterization laboratory. Upon arrival at the emergency department, the patient exhibited restlessness and cold extremities. Blood pressure and heart rate decreased to 57/42 mmHg and 40 bpm, respectively. A repeat ECG confirmed persistent ST-segment elevation myocardial infarction (STEMI) (Figure 1). Notably, ST-segment elevation was also observed in leads V1-V3, suggestive of a concurrent right ventricular infarction.

Regarding the rhythm, distinct P-waves were not clearly discernible, suggesting atrial standstill or a junctional escape rhythm secondary to SA node ischemia. This severe bradyarrhythmia was identified as the primary cause of the hemodynamic collapse.

Diagnostic investigations and intervention

**Figure 2:**
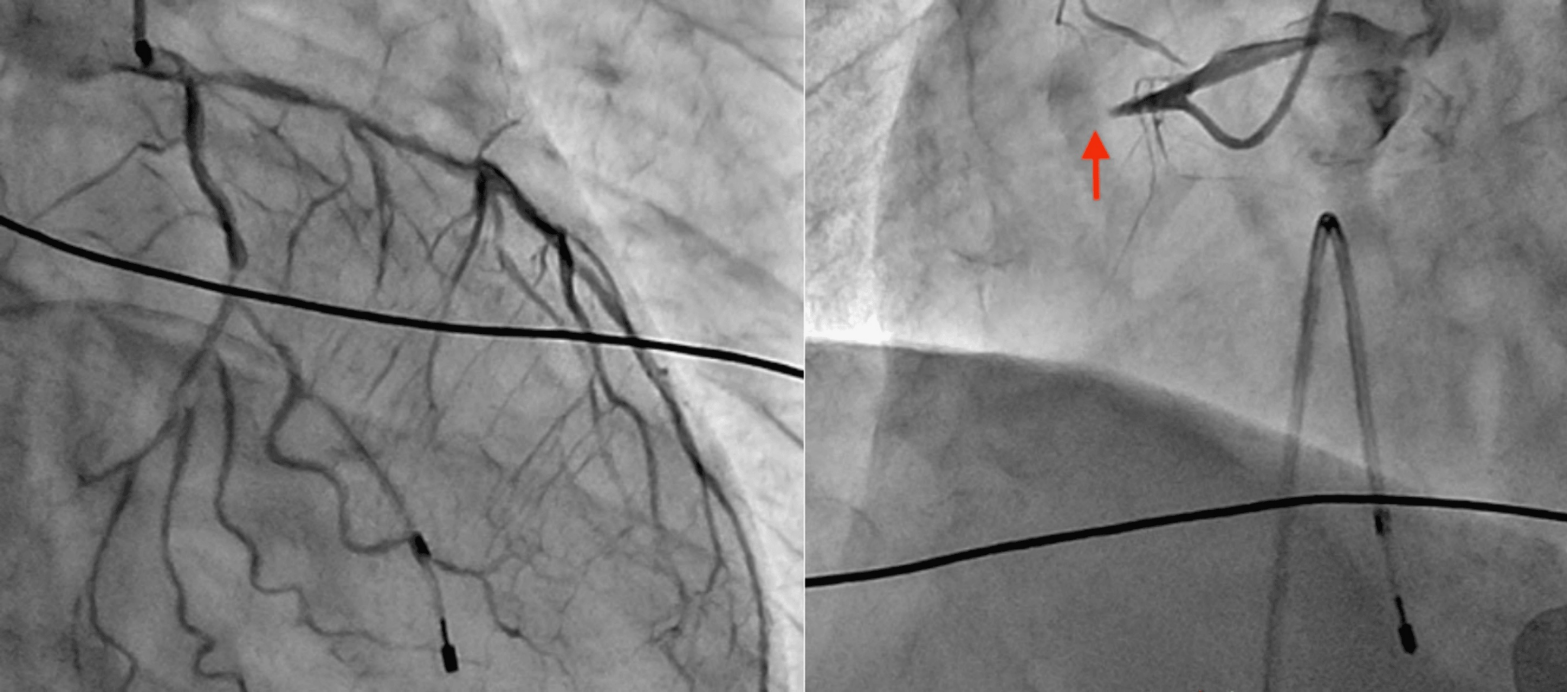
Emergency coronary angiography (CAG). It shows 100% total occlusion of the right coronary artery (RCA segment #1). In addition to the culprit lesion, atherosclerotic stenosis was observed in the left anterior descending (LAD) and left circumflex (LCX) arteries.

**Figure 3:**
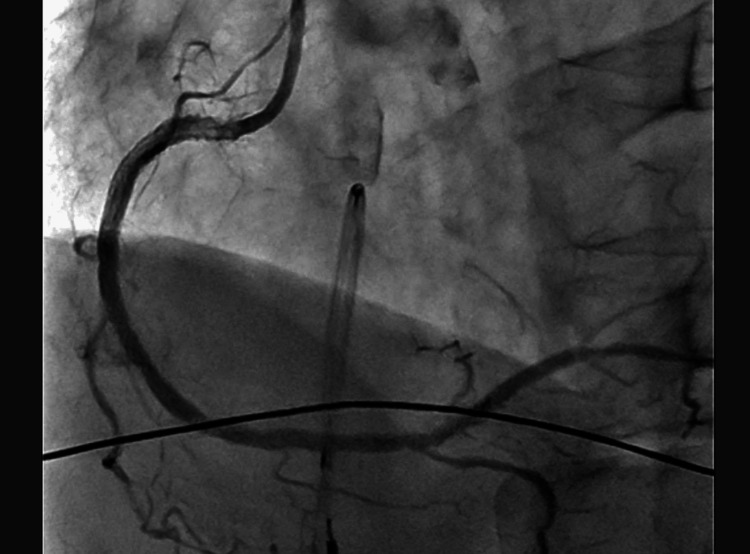
Post-procedural coronary angiography. It shows successful restoration of flow in the right coronary artery after intracoronary stent implantation.

The patient was transferred to the catheterization laboratory 33 minutes after arrival at the hospital. Emergency coronary angiography (CAG) revealed 100% occlusion of the proximal right coronary artery (RCA, segment #1) (Figure 2). Based on the underlying atherosclerotic changes and acute occlusion during anaphylaxis, the patient was diagnosed with Type II KS. Emergency percutaneous coronary intervention (PCI) was performed with the successful implantation of an intracoronary stent (Figure 3). The door-to-balloon time (DTBT) was 46 minutes.

Hospital course and outcome

Following the procedure, the patient was admitted to the intensive care unit (ICU) for hemodynamic monitoring and management of the remaining anaphylactic effects. The clinical condition stabilized, and the patient was transferred to a general ward. He was discharged on the 10th hospital day without residual cardiac complications. Informed consent was obtained for the publication of this case report.

## Discussion

Definition, pathophysiology, and classification

KS, first described by Nicholas Kounis and Zavras in 1991, is defined as the coincidental occurrence of acute coronary syndrome, ranging from vasospastic angina to acute myocardial infarction, triggered by a hypersensitivity reaction [1]. KS results from the release of inflammatory mediators during mast cell degranulation. These mediators act on coronary vascular smooth muscle to induce contraction and can promote the degradation of the fibrous cap of atherosclerotic plaques through the action of proteases such as chymase and tryptase [5].

The syndrome is classified into three primary variants [2]: Type I occurs in patients with normal coronary arteries, where vasospasm is the primary cause of ischemia. Type II occurs in patients with pre-existing coronary artery disease, where the allergic insult triggers plaque rupture or erosion. Type III involves stent thrombosis (Type IIIA) or restenosis (Type IIIB) due to hypersensitivity. In this case, the acute RCA occlusion in a patient with underlying atherosclerosis is consistent with Type II KS.

Incidence

Determining the precise incidence of KS remains challenging due to significant underdiagnosis and the absence of specific ICD-10 codes, leading many cases to be categorized simply as "anaphylactic shock" or "myocardial infarction of unknown cause." However, its clinical relevance is becoming increasingly recognized. A large-scale analysis of U.S. inpatient data (2007-2014) revealed that among 235,420 patients admitted for allergic reactions or anaphylaxis, approximately 1.1% (2,616 patients) developed concurrent acute coronary syndrome, qualifying for a diagnosis of KS [2].

Causative agent

Drugs are the most frequent trigger for KS, accounting for approximately 40% to 50% of all reported cases [2]. While antibiotics and NSAIDs are common triggers [6], analgesics like acetaminophen are less frequently implicated. Vu et al. reported a case of KS presenting as coronary artery spasm associated with acetaminophen infusion, highlighting that even widely used analgesics can precipitate this syndrome [3]. In the current case, the temporal relationship between acetaminophen administration and symptom onset strongly suggests a causal link.

Diagnostic challenges

Diagnosing KS requires a "high index of suspicion," as the simultaneous presentation of allergic and cardiac symptoms can be misleading. A significant diagnostic challenge is the "Kounis paradox," where the expected tachycardia of anaphylaxis is replaced by bradycardia due to the Bezold-Jarisch reflex, particularly in cases involving the RCA. Serum tryptase is considered the gold standard biomarker for mast cell degranulation. However, its short half-life necessitates sampling within a narrow window (30 minutes to a few hours after onset), which often leads to missed diagnoses in emergency settings. In Type II KS, troponin elevation is a key differentiator from Type I, necessitating an invasive evaluation to assess plaque stability.

Therapeutic challenges

The management of KS involves a conflict between anaphylaxis and ischemia protocols. While essential for treating anaphylaxis, epinephrine can worsen coronary vasospasm and increase myocardial oxygen demand via α-adrenergic and β-adrenergic stimulation, potentially aggravating the infarct. Epinephrine is a lifesaving medication in patients with anaphylactic shock. However, physicians should recognize this infarctive complication in patients with risk factors for KS. Conversely, vasodilators used for acute coronary syndrome may exacerbate the systemic hypotension associated with anaphylactic shock. In this case, the administration of epinephrine was required for hemodynamic stabilization but possibly contributed to the subsequent coronary event.

The role of the RRC

The operational structure of the RRC at the treating institution involves a physician-staffed ground emergency medical service established in April 2015. The team is dispatched directly to the patient’s location to assess the condition and contact in-hospital specialists before arrival. This system involves EMS personnel, field emergency physicians, nurses, and cardiologists. Previous research by the authors' group indicated that while DTBT might not always be statistically shortened [7], the RRC enables safe transport and early intervention. The intervention of the RRC provided several clinical advantages in this case. First, the team performed a pre-hospital ECG immediately upon the onset of chest pain, facilitating the pre-activation of the catheterization laboratory. Second, the presence of a physician enabled real-time management of the therapeutic conflict between vasopressor administration and coronary perfusion. Finally, the seamless transition from field intervention to the catheterization laboratory ensured continuous care, achieving a DTBT of 46 minutes.

## Conclusions

This report describes a rare case of Type II KS secondary to acetaminophen-induced anaphylaxis. The case underscores two critical lessons for emergency medicine. First, the RRC system proved instrumental in providing early intervention, allowing for the immediate recognition of cardiac involvement following the onset of anaphylaxis. The presence of a physician in the pre-hospital setting enabled the management of complex hemodynamics that standard EMS protocols might not cover.

Second, emergency medical personnel must maintain a high level of clinical suspicion for KS in any patient presenting with anaphylaxis who develops chest pain or hemodynamic instability disproportionate to the allergic reaction. Since the management requires a delicate balance between treating the allergic reaction and addressing myocardial ischemia, early diagnosis and an integrated system of care are paramount to improving patient outcomes.
